# Twofold optical display and encryption of binary and grayscale images with a wavelength-multiplexed metasurface

**DOI:** 10.1515/nanoph-2023-0324

**Published:** 2023-09-14

**Authors:** Xiaoyi Zhang, Jiaqi Cheng, Wenjing Yue, Zhancheng Li, Duk-Yong Choi, Yang Li, Hongliang Li, Sang-Shin Lee, Shuqi Chen, Song Gao

**Affiliations:** School of Information Science and Engineering, Shandong Provincial Key Laboratory of Network Based Intelligent Computing, University of Jinan, Jinan 250022, China; School of Physics and TEDA Institute of Applied Physics, Nankai University, Tianjin 300071, China; Laser Physics Centre, Research School of Physics, Australian National University, Canberra, ACT 2601, Australia; School of Microelectronics, Shandong University, Jinan 250101, China; Department of Electronic Engineering, Nano Device Application Center, Kwangwoon University, Seoul 01897, Republic of Korea

**Keywords:** dual-wavelength metasurface, twofold optical display and encryption, binary meta-image, grayscale meta-image, quarter-wave plate metasurface

## Abstract

The remarkable capability in regulating light polarization or amplitude at the nanoscale makes metasurface a leading candidate in high-resolution image display and optical encryption. Diverse binary or grayscale meta-images were previously shown concealed in a single metasurface, yet they are mostly stored at same encryption level and share an identical decryption key, running the risk of exposing all images once the key is disclosed. Here, we propose a twofold optical display and encryption scheme demonstrating that binary and grayscale meta-images can be concurrently embedded in a nonspatially multiplexed silicon metasurface, and their decryptions demand for drastically different keys. Unlike previous metasurfaces relying on isolated transmission or phase manipulations upon orthogonal linear polarization incidences, this is made possible by exploiting silicon meta-atoms featuring joint transmission amplitude and polarization control at two wavelengths. In detail, the selected two meta-atoms exhibit large polarization-independent transmission difference (∼85 %) at a wavelength of 800 nm, while functioning as the nano-quarter-wave plate at wavelength of 1200 nm. Through elaborate design in simulation, a binary image can be witnessed when the metasurface is merely illuminated by an unpolarized light of wavelength 800 nm or under white light illumination. However, a distinct binary or grayscale image will come into view by inspecting the metasurface with an analyzer and when the incident light is circularly polarized at the wavelength of 1200 nm. Two metasurface samples are fabricated and successfully verified the claims experimentally. The proposed approach is expected to bring new insights to the field of optical display and encryption.

## 1 Introduction

Optical metasurface is an emerging artificial two-dimensional metamaterial that can arbitrarily manipulate light properties at subwavelength scales by elaborately designing its constituent nanostructures, so-called meta-atoms. Thus, unlike conventional optical elements relying on complex curved shapes and the phase accumulation effect through light propagation over a bulky medium, nanostructure-imparted phase discontinuity makes the metasurface easily tailor light wavefront through an interface. As a result, this salient feature has been successfully applied in realizing diverse optical wave manipulation phenomena such as anomalous beam deflection [1–3], light focusing [4–6], vortex beam generation [7–10], and so on, opening a new avenue for the development of planar optics.

Apart from the regulation of light wavefront based on phase modulation, the nanostructures can also be utilized to manipulate light amplitude and polarization. In consideration of their ultra-tiny size, metasurfaces have shown bright prospects in realizing high-resolution meta-image display, high-capacity information storage, and optical encryption [11–23]. In general, Malus’ law is the common strategy adopted in metasurface-based optical encryption and image display, where each nanostructure is functionally a nano-half-wave plate (nano-HWP) or nanopolarizer with the polarization conversion and polarization-dependent light intensity modulation function, respectively. As such, by elaborately designing the rotation angle of each nanostructure, a spatially varying light polarization or intensity profile can be formed. Particularly for the former case, an individual grayscale image will be disclosed when an analyzer is applied to the output light [24–28]. In addition to hiding a grayscale image in the output light polarization profile, double or multi-image displays and encryptions could also come true in a single metasurface, which mostly utilize the multiplexing technology. Multiplexing is an effective manner to widening the functions and applications of the metasurfaces. Most works realize the simultaneous encryptions of binary and grayscale images into a single metasurface by considering polarization of incidence as the multiplexing channel [29–32]. For example, based on the orientation degeneracy principle, one-to-more mapping of the nanostructure orientation angle and the transmission intensity can be anticipated. With proper design, several binary or grayscale images can be encoded in a metasurface, and these images could be witnessed by selecting proper polarization state of the input and output light through adjusting the polarizer and analyzer. Apart from polarization, wavelength serves as another promising means for optical encryption and image display [33–37]. Lots of works for wavelength-multiplexed metasurface adopt bilayer or interleaved structure configuration, which undoubtedly increases the design complexity and difficulty. For the above-mentioned optical encryption strategies, the binary or grayscale images are mostly found hidden at the same encryption level. In other words, the meta-images can be decrypted using the same measurement setup by continuously adjusting the analyzer polarization angle or bandpass tunable filter. Recently, a new strategy has been introduced into a nanopolarizer-based Malus metasurface design for the purpose of enhancing encryption security [38]. It shows that the acquisition of the real information needing an additional analyzer compared to the acquisition of the camouflage information. Because of this difference, the two binary images are said to be stored at different encryption levels.

In this work, we propose and demonstrate a wavelength-multiplexed dielectric metasurface for twofold optical display and encryption of both binary and grayscale images. Specifically, we have systematically investigated the transmission amplitude and polarization responses of the dielectric meta-atom at two wavelengths. Two meta-atoms exhibiting giant transmission amplitude differences at the wavelength *λ*_1_ = 800 nm while both functioning as the nano-quarter-wave plate (nano-QWP) at the wavelength *λ*_2_ = 1200 nm are selected. Through elaborate engineering of the orientation angles of the spatially distributed meta-atoms, a polarization-insensitive binary image can be seen when a narrowband optical filter is inserted before the metasurface, whereas another different grayscale image will be revealed in a higher encryption level, when a distinct optical filter, polarizer, quarter-wave plate, and analyzer are simultaneously applied to the optical path. Due to the use of two meta-atoms, the binary image designed for *λ*_1_ can also be colorized under white light illumination, yet the image designed for *λ*_2_ can barely be seen from the former two cases. Therefore, while the metasurface can display an optical image under white light illumination, with a few decryption keys, identical image can also be witnessed under *λ*_1_ and will firmly convince the public that this is the “real” information. Nevertheless, the exactly real image is designed for *λ*_2_ and must be decrypted with a variety of different components, thus ensuring a highly enhanced security level. Compared with previous wavelength-multiplexed metasurfaces resorting to bilayer or interleaved nanostructures, we developed a noninterleaved single-layer metasurface to respond to diverse wavelength to simplify the design process. We anticipate that the proposed metasurface with wavelength-selected transmission amplitude and polarization responses could provide new insight into optical display encryption and be of interest to the relevant field.

## 2 Results and discussion

### 2.1 The schematic of the proposed metasurface and the selection of the nanopost

**Figure 1:**
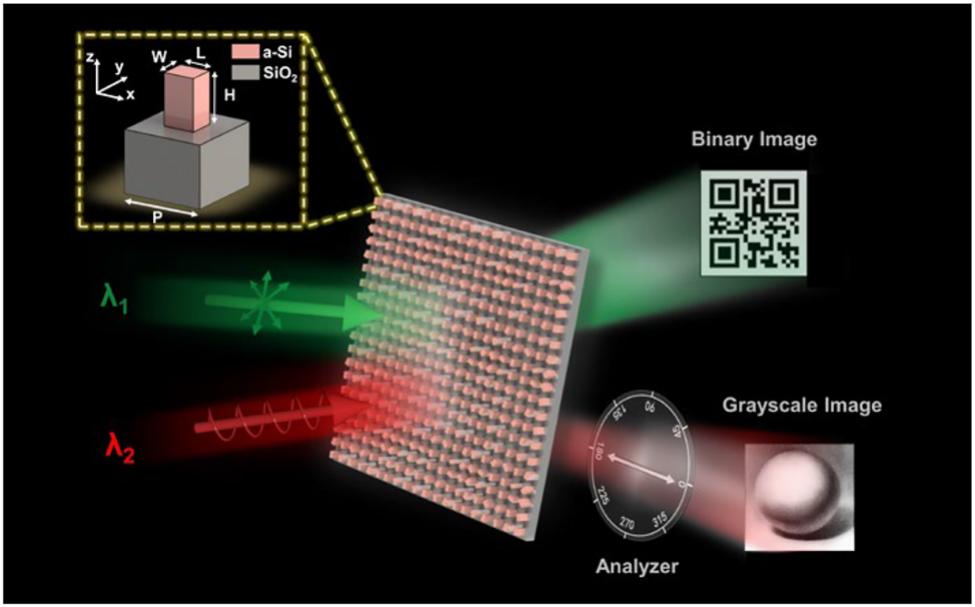
Schematic illustration of the proposed twofold optical display and encryption of binary and grayscale images based on the double-wavelength dielectric metasurface. The incident light at wavelength *λ*_1_ will disclose a binary image. A grayscale image can be revealed only when the incident light is at wavelength *λ*_2_ and circularly polarized, with a proper analyzer being placed behind the metasurface. The top left corner depicts the structure diagram of the unit cell containing a rectangular a-Si nanopost atop a SiO_2_ substrate.

Figure 1 shows the schematic of the proposed wavelength-multiplexed metasurface for optical encryption with different decryption difficulties. Considering that most works on metasurface-based image display and encryption are demonstrated in the visible band. We choose two wavelengths in the near-infrared region from the point view of encryption since it is invisible to human eyes. When the metasurface is illuminated by light of wavelength *λ*_1_, the transmitted light will form a binary intensity pattern. In contrast, a distinct grayscale image will appear when the light wavelength is changed to *λ*_2_ with circular polarization and a proper analyzer is applied in the optical path. The green and red colors are just for indicating that different wavelengths are used in the near-infrared region. It shows a giant difference in optical elements used for decrypting the two meta-images, thus implying a highly enhanced encryption security level. Note that the proposed design strategy also holds great promise in carrying camouflage information as the binary image can be specially designed as a QR code pattern to intentionally confuse the public by linking to the fake information.

We give a demonstration of the twofold optical display and encryption concept in the near-infrared region using an all-dielectric metasurface based on amorphous silicon (a-Si), considering it features less absorptive for red light and even lossless in the near-infrared region. The unit cell of the metasurface is illustrated in Figure 1, where a rectangular a-Si nanopost is placed on a silica substrate. The unit cell is square-shaped, with a periodicity *P* = 400 nm that is much smaller than the operation wavelengths of *λ* = 800 nm and 1200 nm, therefore, following the Nyquist sampling criterion and high order diffractions can be prohibited [39, 40]. The nanopost is also designed to be subwavelength thick (*H* = 500 nm). The periodicity and nanopost thickness are the optimized results following our previous work on a-Si metasurfaces, by taking account of the same ratio factors of periodicity and nanopost thickness divided by the operating wavelength [4, 41].

**Figure 2:**
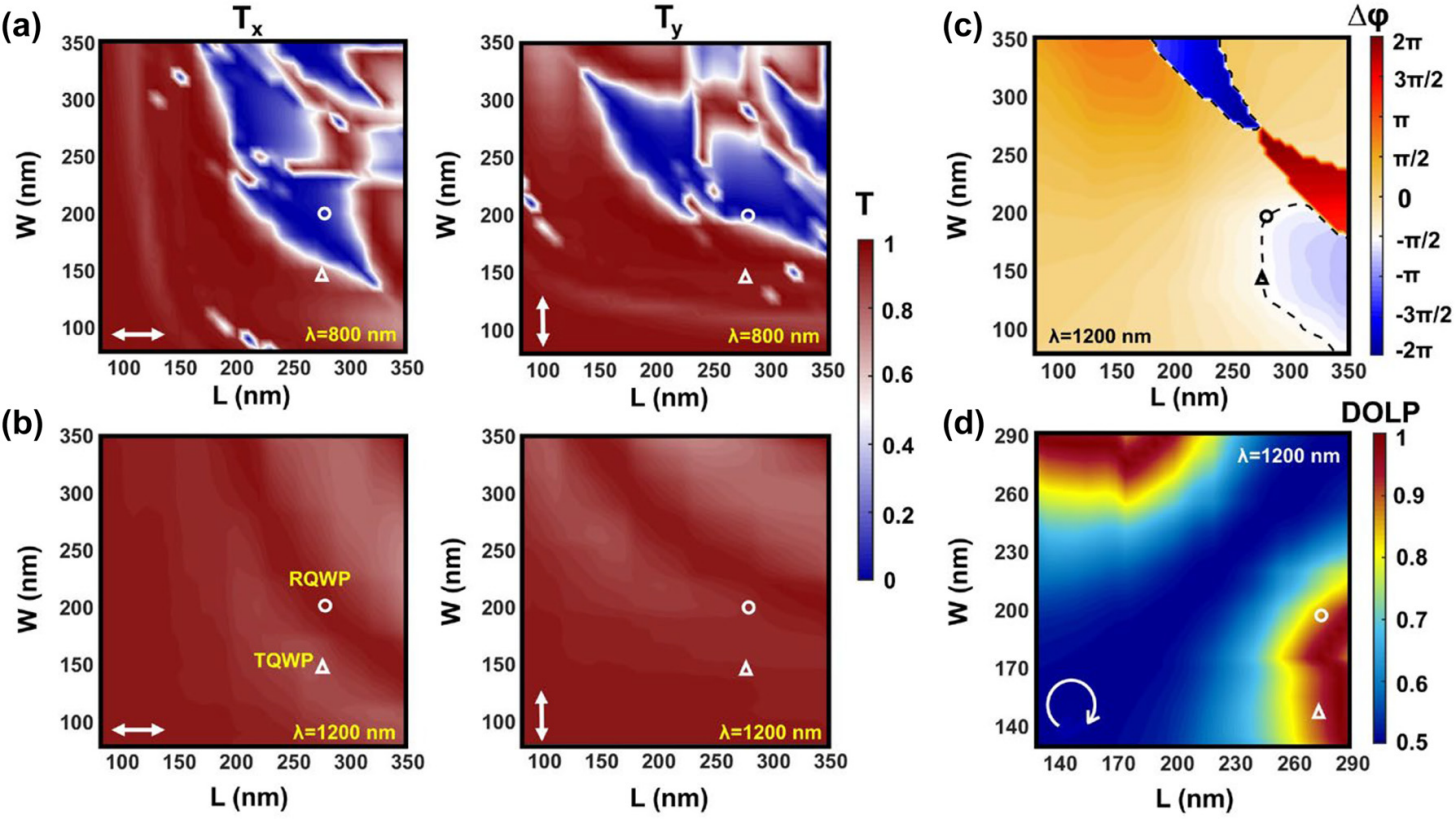
Simulated transmission as a function of the meta-atom length and width under *x*- and *y*-polarizations at wavelength (a) 800 nm and (b) 1200 nm. (c) Simulated phase difference Δ*φ* = *φ*_*y*_ − *φ*_*x*_ at 1200 nm, where *φ*_*x*_ and *φ*_*y*_ are the phase for incident *x*- and *y*-polarizations, respectively. The black dashed line represents the contour of −*π*/2. (d) Simulated DOLP with respect to the length and width of meta-atom at 1200 nm.

Unlike previous metasurfaces whose meta-atoms are merely equivalent to nano-HWP for linear polarization rotation at one single wavelength, the proposed concept is fulfilled by considering the transmission amplitude at *λ* = 800 nm and polarization conversion at *λ* = 1200 nm. More specifically, two meta-atoms that separately exhibit near-unity and zero transmission efficiency at *λ* = 800 nm and concurrently function as the nano-QWP at *λ* = 1200 nm are expected to realize the binary and grayscale images, respectively. Subsequently, numerical simulations based on the finite-difference time-domain method are carried out to find the desired meta-atoms. Figure 2(a) shows the swept transmission efficiencies (*T*_*x*_ and *T*_*y*_ under *x*- and *y*-polarizations, respectively) as a function of the meta-atom’s length (*L*) and width (*W*) from 80 nm to 350 nm for incident light at *λ* = 800 nm. It can be seen from the results that meta-atoms with high and low transmission efficiency can be achievable under this wavelength. Similarly, the transmission efficiencies at *λ* = 1200 nm are examined as depicted in Figure 2(b). In this case, besides the high transmission efficiency, a phase difference of ±*π*/2 between *x*- and *y*-polarization (*φ*_*x*_ and *φ*_*y*_) is mandatory for meta-atoms working as the QWP. As such, the corresponding phase difference Δ*φ* = *φ*_*y*_ − *φ*_*x*_ is also numerically investigated as presented in Figure 2(c).

Considering that binary and grayscale images need to be embedded in a single-layer nonspatially multiplexed metasurface, the selected meta-atoms must simultaneously meet the demands required under the two wavelengths. Based on the above analysis, two meta-atoms are selected as marked by the white triangle and circle in Figure 2(a)–(c). For simplicity, we will name the meta-atoms as TQWP (triangle) and RQWP (circle), respectively, where the first letter denotes high or low transmissions at *λ* = 800 nm (“T” relates to high transmission, and “R” is associated to low transmission corresponding to high reflection, which will be evidenced later) and QWP represents the polarization conversion behavior at *λ* = 1200 nm. The geometric parameters of TQWP are *L* = 275 nm, *W* = 145 nm, and that for RQWP are *L* = 276 nm, and *W* = 200 nm. Note that a pioneering work also utilizes two types of meta-atoms for metasurface design, which yet mainly focuses on the decoupling of multiple light characteristics at a single wavelength and increasing the function integrations [42]. In our work, the average transmissions of selected TQWP and RQWP are 92 % and 7 %, respectively, giving rise to a transmission difference of 85 % at *λ* = 800 nm. And the two meta-atoms exhibit a similar orthogonal linear polarization phase difference of Δ*φ* = −*π*/2 at *λ* = 1200 nm. Such functional diversity under multi-wavelengths could provide a novel manner for enlarging the difference between keys used in decrypting different images. To further verify that the two meta-atoms act as nano-QWP at this wavelength, additional simulations are performed. To further verify that the two meta-atoms act as nano-QWP at this wavelength, additional simulations are performed. For a QWP, it is expected that an incident light with circular polarization will be transformed into linear polarization, while the linear polarization can only be converted to circular polarization under specific conditions, i.e., when the intersection angle between QWP axis and linear polarization is ±45°. In the former case, the converted linear polarization can be quantitatively evaluated by the degree of linear polarization (DOLP) expressed as DOLP = 1 − 2*R*/(1 + *R* ^2^), where *R* is the length ratio between major and minor axes associated with the ellipse [41]. The output light will be more likely linearly polarized when the DOLP is approaching 1. Figure 2(d) shows the corresponding simulated DOLP as a function of meta-atom’s length and width under right-handed circular polarization (RCP) light incidence at *λ* = 1200 nm. To reduce the simulation time, the length and width ranges are slightly narrowed in the vicinity of the TQWP and RQWP. It can be seen from Figure 2(d) that the DOLP of transmitted light for the selected TQWP and RQWP are approximately 1, implying that they could be treated as quasi-perfect nano-QWPs at *λ* = 1200 nm. It should be noted that although meta-atoms with lengths around 290 nm promise relatively higher DOLP, they are not suitable to be selected since the transmission difference at *λ* = 800 nm is not sufficient to guarantee a high-quality binary image.

### 2.2 Theoretical analyses and numerical results showing the effect of the meta-atoms’ orientation angle on transmission and polarization conversion

**Figure 3:**
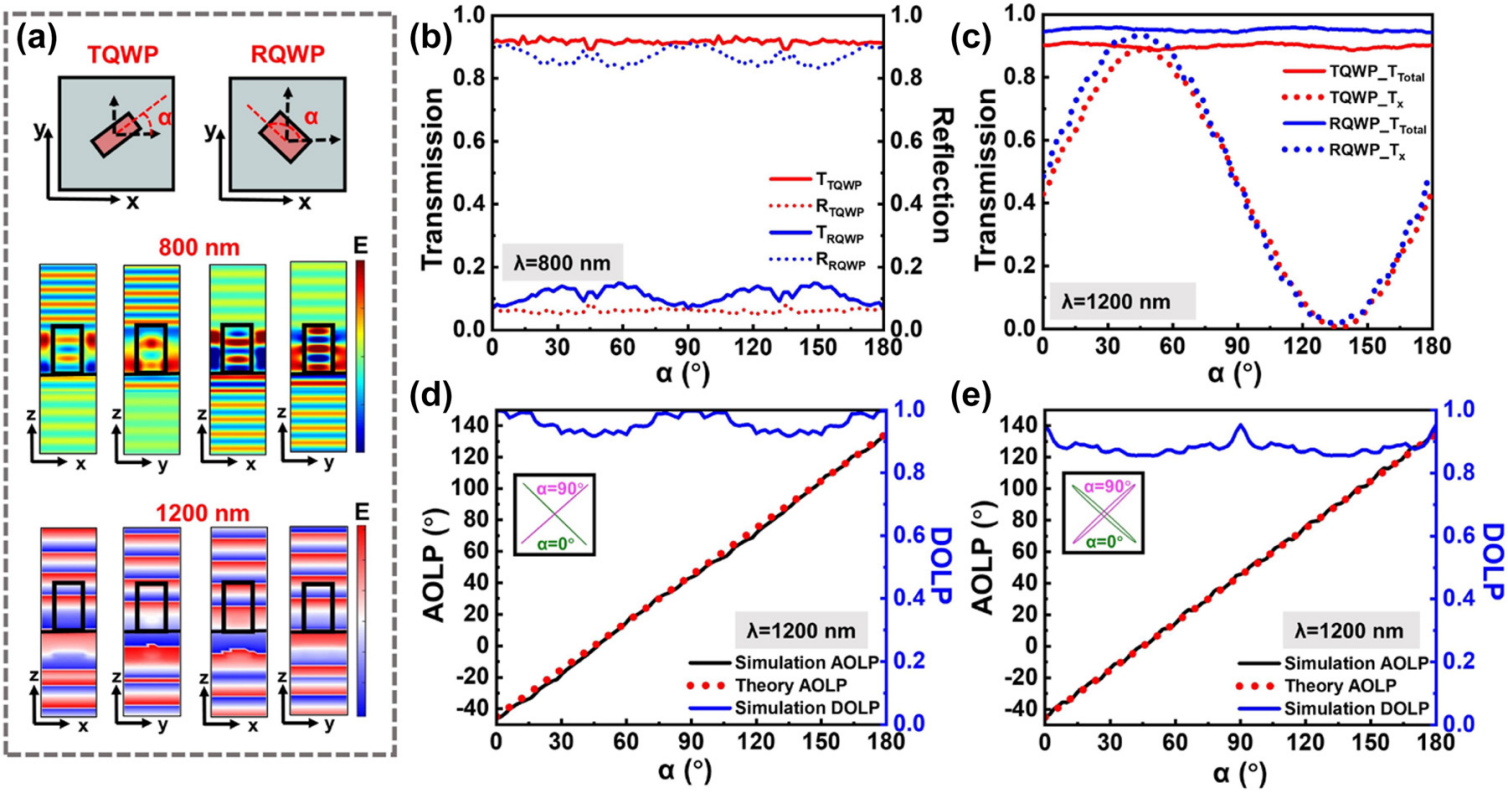
Numerical simulation results of selected TQWP and RQWP. (a) Top view of the TQWP and RQWP schematics. The simulated cross-sectional electric field for TQWP and RQWP at wavelength 800 nm and phase wavefront at wavelength 1200 nm under *x*- and *y*-polarizations, respectively. (b) The simulated total transmission and reflection as a function of various TQWP and RQWP orientation angles under RCP incidence at 800 nm. (c) The simulated total and *x*-polarized transmission with respect to different orientation angles under RCP incidence at 1200 nm. The DOLP and AOLP in simulation or theory as a function of the orientation angle for (d) TQWP, and (e) RQWP at wavelength 1200 nm. (Insets are the simulated polarization ellipse maps when the orientation angle of meta-atom is 0° and 90°).

The large polarization-insensitive transmission difference rendered by the as-selected meta-atoms at *λ* = 800 nm is indispensable for realizing high-quality binary image and can be attributed to the highly transmissive and reflective responses of the TQWP and RQWP, respectively, in view of the negligible material absorption loss. As shown in Figure 3(a), the simulated cross-sectional electric fields for the case of the initial TQWP and RQWP (with orientation angle *α* = 0°) under *x*- and *y*-polarizations are proven to be highly transmissive and reflective. Here, the orientation angle *α* is defined as the angle between the slow axis of the meta-atoms and *x*-axis. The transmitted wavefronts at *λ* = 1200 nm for unrotated TQWP and RQWP are also provided, where near *π*/2 phase delay between *x*- and *y*-polarizations can be observed for both meta-atoms. While a binary image can be easily implemented at *λ* = 800 nm by simply relying on the high and low transmissions using the unrotated TQWP and RQWP only, for realizing the grayscale image at *λ* = 1200 nm, it is inevitable to have the meta-atoms rotated for generating specific linear polarization profiles. Therefore, the effect of the meta-atoms’ orientation angle on the performances at the designed wavelengths is systematically investigated. Light incidence with RCP is directly used in the simulation since it carries both *x*- and *y*-polarization components and can simplify the analysis without biasing the results. It is presented in Figure 3(b) and (c) that at *λ* = 800 nm, the transmissions of TQWP and RQWP are maintained at high and low levels with average values of ∼94 % and ∼20 %, respectively, for the orientation angle *α* ranging from 0° to 180° in a step of 2°. This near 74 % transmission difference implies that TQWP and RQWP with any orientation angle will not significantly influence the quality of binary image. For the case of *λ* = 1200 nm, both TQWP and RQWP are highly transmissive in the investigated orientation angle range, with comparable average total transmissions of 89.4 % and 94.6 %, respectively, as shown in Figure 3(c). While the indistinguishable total transmission of TQWP and RQWP can hardly be used in optical encryption, continuous sinusoidal transmission intensity modulations can be achieved if only the *x*-polarization component is considered (dotted lines in Figure 3(c)). It is believed that the input RCP is transformed into different linear polarizations with respect to various orientation angles. Figure 3(d) presents the simulated DOLP and angle of linear polarization (AOLP) of transmitted light as a function of the orientation angle of TQWP. The blue solid line demonstrates that the DOLP slightly deviates from the ideal value of 1 but is still higher than 0.92. Theoretical analysis is then conducted for the AOLP. It is known that the Jones matrix of an ideal QWP with the slow axis along the *x*-axis can be expressed as: *M*_0_ = $\left[\begin{array}{cc} 1 & 0\\ 0 & -\text{i} \end{array}\right]$ . When it is rotated with an angle *α*, the Jones matrix can be revised to be:

(1) $$\begin{array}{c} \;M_{1}=R\left(-\alpha \right)\cdot M_{0}\cdot R\left(\alpha \right)\\ =\left[\begin{array}{cc} \cos\alpha & -\sin\alpha \\ \sin\alpha & \cos\alpha \end{array}\right]\left[\begin{array}{cc} 1 & 0\\ 0 & -\text{i} \end{array}\right]\left[\begin{array}{cc} \cos\alpha & \sin\alpha \\ -\sin\alpha & \cos\alpha \end{array}\right]\\ =\left[\begin{array}{cc} \text{co}{\text{s}}^{2}\alpha -\text{isi}{\text{n}}^{2}\alpha & \cos\alpha \sin\alpha +\text{icos}\alpha \sin\alpha \\ \cos\alpha \sin\alpha +\text{icos}\alpha \sin\alpha & \text{si}{\text{n}}^{2}\alpha -\text{ico}{\text{s}}^{2}\alpha \end{array}\right] \end{array}$$

For an RCP incidence with a Jones vector of *J*_0_ = $\frac{1}{\sqrt{2}}\left[\begin{array}{c} 1\\ -\text{i} \end{array}\right]$ , the light transmitted through the QWP is derived as:

(2) $$\begin{array}{ll} J_{1} & =M_{1}\cdot J_{0}\\ & =\frac{1}{\sqrt{2}}\\ & \;\times \left[\begin{array}{cc} \text{co}{\text{s}}^{2}\alpha -\text{isi}{\text{n}}^{2}\alpha & \cos\alpha \sin\alpha +\text{icos}\alpha \sin\alpha \\ \cos\alpha \sin\alpha +\text{icos}\alpha \sin\alpha & \text{si}{\text{n}}^{2}\alpha -\text{ico}{\text{s}}^{2}\alpha \end{array}\right]\\ & \;\times \left[\begin{array}{c} 1\\ -\text{i} \end{array}\right]\\ & =\left[\begin{array}{c} \cos\left(\alpha -\frac{\pi }{4}\right)\\ \sin\left(\alpha -\frac{\pi }{4}\right) \end{array}\right]{\text{e}}^{-\text{i}\alpha } \end{array}$$

The result (2) shows that the transmitted light is linearly polarized with an AOLP of $\alpha -\frac{\pi }{4}$ , which is closely related to the orientation angle of QWP. Thus, continuous modulation of light intensity can be accomplished by considering a polarization component and applying the Malus’ law. Similar process is conducted for left-handed circular polarization (LCP) incidence case, where the AOLP is $\alpha +\frac{\pi }{4}$ . For the TQWP, the simulated AOLP (black solid line) is in excellent agreement with the theoretical one (dashed red line in Figure 3(d)). The insets depict two polarization ellipse maps associated with the TQWP of orientation angle 0° and 90°. The simulated DOLP and AOLP for the case of RQWP are also provided in Figure 3(e), which show similar trend to that of the TQWP. It can be found that for the same orientation angle, the linearity of transmitted light polarization in TQWP is slightly higher than that of RQWP. All in all, with rotation of the TQWP or RQWP, arbitrary modulation of polarized light intensity ranging from 0 to near unity can be achieved and thus a grayscale image could be embedded in the metasurface at *λ* = 1200 nm.

### 2.3 The simulation and experiment results in twofold display and encryption of binary and grayscale images

Based on the selected meta-atoms, we first verify the optical encryptions of two binary images as have been done by previous works. The metasurface sample is designed to be 40 μm × 40 μm in area, corresponding to 100 × 100 meta-atoms. The meta-image is a QR code for *λ* = 800 nm and that for *λ* = 1200 nm is a butterfly. For concrete design of the metasurface, the intensity in each pixel of the QR code determines which type of meta-atom (TQWP or RQWP) should be used, while the intensity in butterfly decides the orientation angle of the meta-atom. For better visualization, one pixel of the QR code is designed to contain 4 × 4 sub-pixels. Based on the above design rule, it can be known that the 16 meta-atoms in each pixel are the same but can have different orientation angles under the joint effect of the dual meta-images. For the construction of the metasurface, the “bright” and “dark” areas in QR code meta-image are filled with TQWP and RQWP, respectively. These meta-atoms will be rotated by 45° and 135° if the corresponding areas in the butterfly meta-image are “bright” and “dark,” respectively.

**Figure 4:**
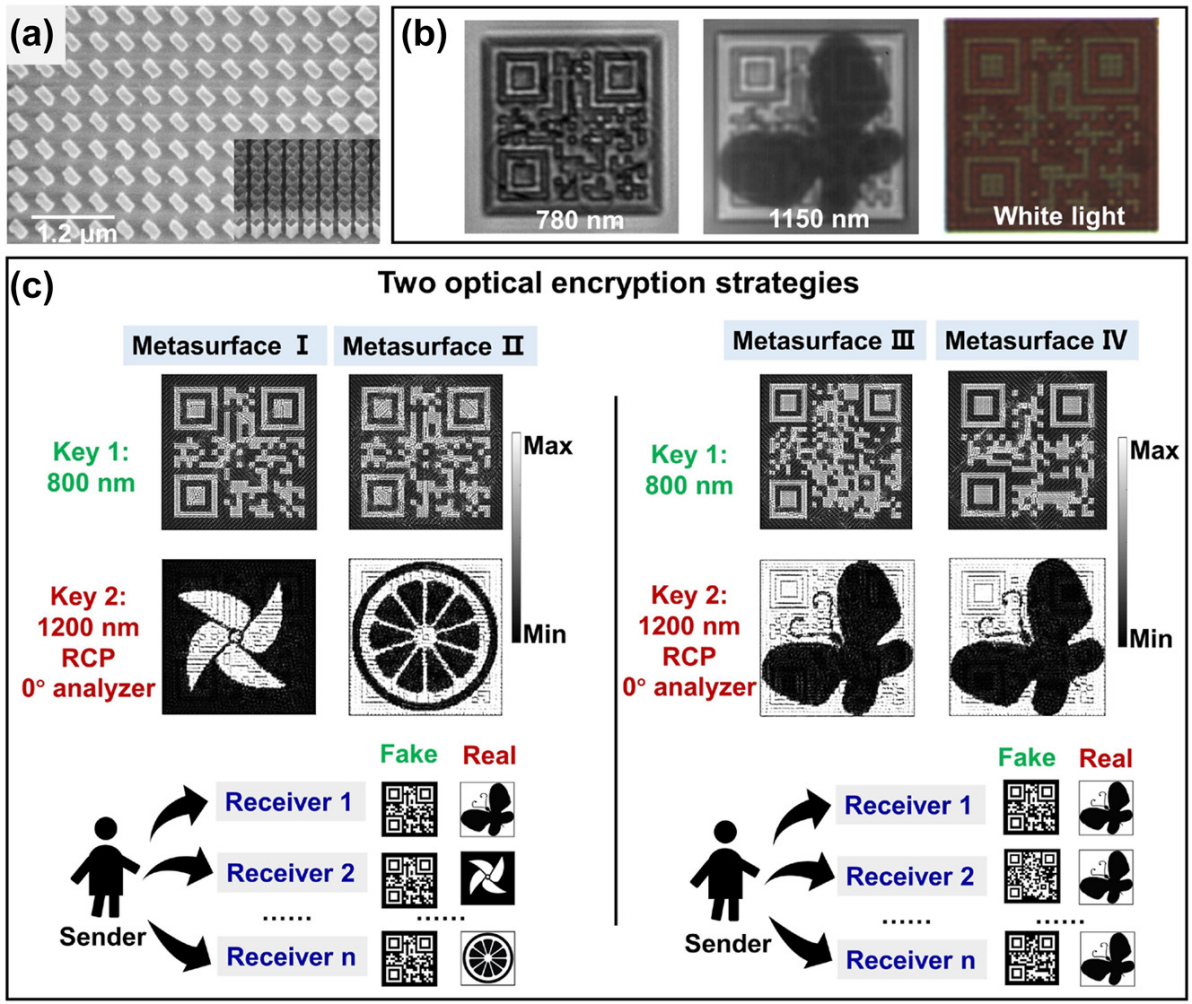
The experimental results and optical encryption strategies. (a) SEM image of the fabricated dual-binary image metasurface. (b) Experimentally obtained optical images under natural white light illumination and at single wavelength incidences of 780 nm and 1150 nm with designed decryption keys. (c) Schematic and simulation results relating to two optical encryption strategies.

Figure 4(a) displays the scanning electron microscopy (SEM) image of the fabricated dual-binary image metasurface. Figure 4(b) is the experimentally obtained optical images under natural white light illumination and at single wavelength incidences of 780 nm and 1150 nm (while the metasurfaces are designed to operate at wavelengths of 800 nm and 1200 nm, due to the limited lab facilities, we only conducted the experiments with the help of available optical filters centered at 780 nm and 1150 nm) with designed decryption keys. Despite the image crosstalk, all the designed images are successfully obtained. The fabrication and measurement details can be found in the Experimental section. Here, all designs are investigated under normal incidence conditions. We further found that the meta-atoms can only hold good performance when the incidence angle is less than 20°. The analysis for the design under oblique incidence angles is shown in Note S1 in Supplementary Material. We notice that the captured QR code image, whose transmission intensity profile is dependent on the type of meta-atom (TQWP or RQWP), shows a low fidelity. The main reason is that the transmission difference of TQWP and RQWP drops below 40 % in the wavelength range from 700 nm to 900 nm except that near the designed wavelength of 800 nm, as provided in Figure S2(b). Therefore, it is expected that high fidelity image can be seen only around the wavelength of 800 nm. Analysis on the effect of transmission difference on image quality is provided in Figure S2. For the meta-image at 1150 nm, the pattern of butterfly could be seen incidence of RCP accompanied by an analyzer with 0° polarization angle in the output light path. Compared with the former case, the crosstalk issue in this case is more severe, which can be understood from multiple aspects, for instance, the mismatched illumination light wavelength and the imperfectly fabricated structures. Also, the abrupt changes in structure size near the boundaries of the image designed for wavelength 800 nm can lead to significant unwanted diffraction and scattering effect and aggravate the crosstalk. Further, the periodicity (*P* = 400 nm) of the current metasurface unit cell is only one-third of the wavelength 1200 nm, which may engage near-field coupling effect between TQWP and RQWP and result in a drastically reduced transmission and further deteriorate the meta-image quality. The detailed analysis about the crosstalk is described in Note S3 in Supplementary Material. Interestingly, since the two designed meta-atoms (TQWP and RQWP) exhibit different transmission spectra in visible band, the appearance of the metasurface under white light illumination will and only resembles the image designed at 800 nm (as shown in Figures 4b and S4). It is also experimentally verified that the image designed at wavelength 1200 nm cannot be revealed under either white light illumination or single wavelength incidence at 800 nm.

As the two meta-images could be designed independently, this dual-wavelength metasurface can be considered as a good platform for optical encryption (at 1200 nm wavelength) carrying a camouflage image (at wavelength 800 nm or under white light illumination). Thus, we propose two optical encryption strategies and correspondingly four metasurfaces (I–IV) are numerically discussed in Figure 4(c). For metasurfaces I and II, they will display the identical QR code image at wavelength 800 nm, but the encrypted image at wavelength 1200 nm could be totally different. Conversely, in metasurfaces III and IV, the QR code image at wavelength 800 nm is different, yet in reality they transmit identical images at wavelength 1200 nm. This demonstrates that the real information from a message sender can only be decrypted by the receiver with correct decryption key. A bystander without correct key only sees the fake information and not able to judge the authenticity.

**Figure 5:**
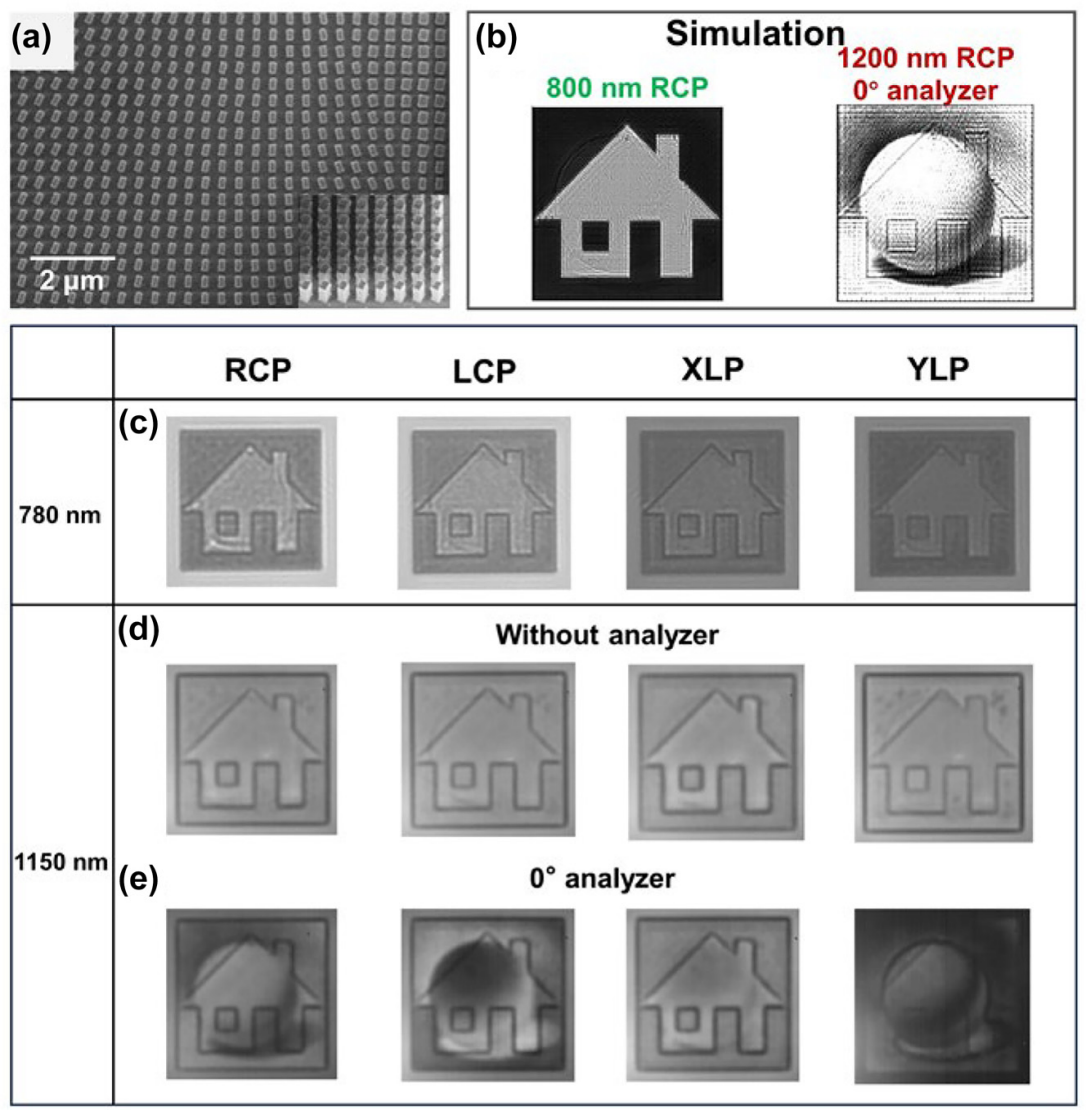
The simulation and experimental results for the binary and grayscale image metasurface. (a) SEM image of the binary and grayscale image metasurface. (b) The simulation results under different input and output conditions. (c) The experimental results showing identical binary images under different polarization incidences at wavelength 780 nm. (d) The experimental results under different polarization incidences at wavelength 1150 nm, obtained without and (e) with a 0° analyzer.

In addition to the binary image, the circular-to-linear polarization conversion capability of the two meta-atoms also hold promise in realizing grayscale image at *λ* = 1200 nm. Toward that goal, we further fabricated a new metasurface incorporating 100 × 100 meta-atoms. The target meta-images to be encoded in the metasurface are a binary house and a grayscale sphere drawing for *λ* = 800 nm and 1200 nm, respectively. The metasurface design rule basically follows the same principle as above discussed in the dual-binary image case. Yet, as the sphere drawing contains random grayscale values between 0 and 1, the orientation of each meta-atom can be arbitrary. In this case, each meta-atom can be regarded as an independent pixel. Figure 5(a) shows the SEM image of the binary and grayscale image metasurface, and Figure 5(b) presents the simulation results under different input and output conditions, which are identical to the dual-binary image metasurface case. Similarly, the simulation results show that the crosstalk mainly exists in the grayscale image carrying real information. For the binary image with fake information, the negligible crosstalk ensures that one cannot extract the real information. Experimental results are further provided in Figure 5(c) through 5(e). In this binary and grayscale image metasurface case, the transmission light intensity profiles under RCP, LCP, *x*-linear-polarization (XLP), and *y*-linear-polarization (YLP) incidences are all examined. For the wavelength of 780 nm, similar house meta-images can be witnessed in all cases due to the polarization-insensitive transmission property of the meta-atoms. When it comes to the wavelength of 1150 nm, our previous theoretical analyses demonstrate that incidences of RCP or LCP will be converted to various linear polarizations following the relationship of *α* − 45° and *α* + 45°, respectively, and quasi-evenly distributed transmission intensities will be expected. For the incidence of XLP or YLP, the versatile meta-atom orientation angles make the output light a mixture of various polarizations including rigorously circular and elliptical polarizations. Therefore, no meta-images can be revealed without analyzer in the output light path (Figure 5(d)). To unveil the designed grayscale sphere image, an analyzer with correct polarization angle is indispensable. As illustrated in Figure 5(e), for incidence of RCP, the grayscale sphere image is displayed when a 0° analyzer is applied. Conversely, a complementary image of the grayscale sphere drawing will be shown under LCP incidence. While ideally no meta-images are expected to be seen for the linear polarization incidences, i.e., the XLP or YLP shall be totally transmitted or blocked when observing the *x*-polarization component. In reality, the generated elliptical polarizations with versatile ellipticities will also project the *x*-polarization component with various efficiencies. The outline of the grayscale image can be slightly seen, but the designed meta-image with high fidelity cannot be obtained. It can still be concluded from the above results that not only one has to change the incident light wavelength but also to set the circular polarization and to insert a 0° analyzer for the purpose to decrypt the real image. Corresponding simulation results can be found in Figure S5 in Supplementary Material. These results successfully prove the feasibility of the scheme for binary and grayscale image encryptions with drastic differences in using optical elements for their display and decryption.

## 3 Conclusions

In summary, we have experimentally demonstrated that monolayer a-Si metasurfaces can be meticulously designed to simultaneously encode a binary and a grayscale image into different display and encryption levels. The constituents of the proposed metasurface include TQWP and RQWP, which are highly transmissive and reflective, respectively, yielding near 85 % transmission difference upon arbitrarily polarized incidence at *λ* = 800 nm and both working as highly transmissive nano-QWPs enabling circular-to-linear polarization conversions at *λ* = 1200 nm. The performances for TQWP and RQWP at two operation wavelengths are well maintained for different meta-atom orientation angles and match well with the theoretical predictions. Based on the TQWP and RQWP, we have proposed a general method to construct the metasurface for twofold optical display and encryption. Numerical and experimental results demonstrate that there’s a giant difference of keys to be used for decrypting the two images. Therefore, the proposed exotic encryption scheme can greatly enhance the freedom and security level of optical encryption. We further envision that the wavelength-multiplexing–assisted twofold image display and encryption scheme will bring new insights and application prospects in multidimensional optical encryption, image display, and high-density information storage.

## 4 Experimental section

### 4.1 Sample preparation

The metasurfaces were prepared with a series of standard nanofabrication techniques including electron beam lithography (EBL), Al etch mask liftoff, and silicon plasma etching. First, plasma-enhanced chemical vapor deposition (Plasmalab 100 from Oxford) was used to deposit the amorphous silicon (a-Si) film on SiO_2_ substrate. Next, a positive electron beam resist (ZEP520A from Zeon Chemicals) was spin-coated on the a-Si film. The metasurface pattern was then formed using an electron beam writer (EBL, Raith150), accompanied by the development in ZED-N50. Subsequently, an Al layer was deposited by the e-beam evaporation (Temescal BJD-2000), and it was patterned by lifting off the resist using a solvent (ZDMAC from Zeon Co.). The patterned Al was used as a hard mask during dry etching, and the designed pattern was transferred to the underlying a-Si film through fluorine-based inductively coupled plasma-reactive ion etching (Oxford Plasmalab System 100). The residual Al etch mask was finally etched in phosphoric/nitric/acetic acids mixed solution.

### 4.2 Optical measurement

The experimental setups for measuring the meta-image in dual-wavelength channel are shown in Figure S6. Incoherent light sources of COPS30-W and GLORIA-T150A are used for white light and single-wavelength measurement, respectively. The light emitting from the light sources successively passes through an aperture, optical filter of the designated wavelength, broadband polarizer (Thorlabs, LPNIR050-MP2), achromatic quarter-wave plate (B. Halle Nachfl), objective lens 1 (Sigma NIR plan apo 10×, NA = 0.3), sample, objective lens 2 (Sigma NIR plan apo 50×, NA = 0.67), and broadband polarizer (Thorlabs, LPNIR050-MP2). Depending on the situation, some of the optical elements may be removed to match the actual illumination conditions. Lastly, for the imaging system, three different cameras are used for capturing the images. The image under white light illumination is captured by CCD (ISH130), while that for wavelength 780 nm is recorded by CMOS (MIchrome 5BW), and lastly the image for 1150 nm is obtained by CCD (C10633-23).

## Supplementary Material

Supplementary Material Details
