# Supplementary material for: Twofold optical display and encryption of binary and grayscale images with a wavelength-multiplexed metasurface
Source: Nanophotonics. 2023 Sep 14;12(19):3747–56. doi: 10.1515/nanoph-2023-0324 (PMC11636522; doi:10.1515/nanoph-2023-0324)
Supplement: Supplementary file 1 — Supplementary Material Details [file j_nanoph-2023-0324_suppl_001.pdf]

Supplemental Material

Twofold optical display and encryption of binary and grayscale images with a wavelength-multiplexed metasurface

Xiaoyi Zhang[1], Jiaqi Cheng[2], Wenjing Yue[1], Zhancheng Li[2]*, Duk-Yong Choi[3], Yang Li[4], Hongliang Li[5], Sang-Shin Lee[5], Shuqi Chen[2]*, and Song Gao[1]**

[1]School of Information Science and Engineering, Shandong Provincial Key Laboratory of Network Based Intelligent Computing, University of Jinan, Jinan 250022, China

[2]School of Physics and TEDA Institute of Applied Physics, Nankai University, Tianjin 300071, China

[3]Laser Physics Centre, Research School of Physics, Australian National University, Canberra ACT 2601, Australia

[4]School of Microelectronics, Shandong University, Jinan 250101, China

[5]Department of Electronic Engineering, Nano Device Application Center, Kwangwoon University, Seoul 01897, Republic of Korea

Note S1

The simulation and measurement results of the design under oblique incidence angles

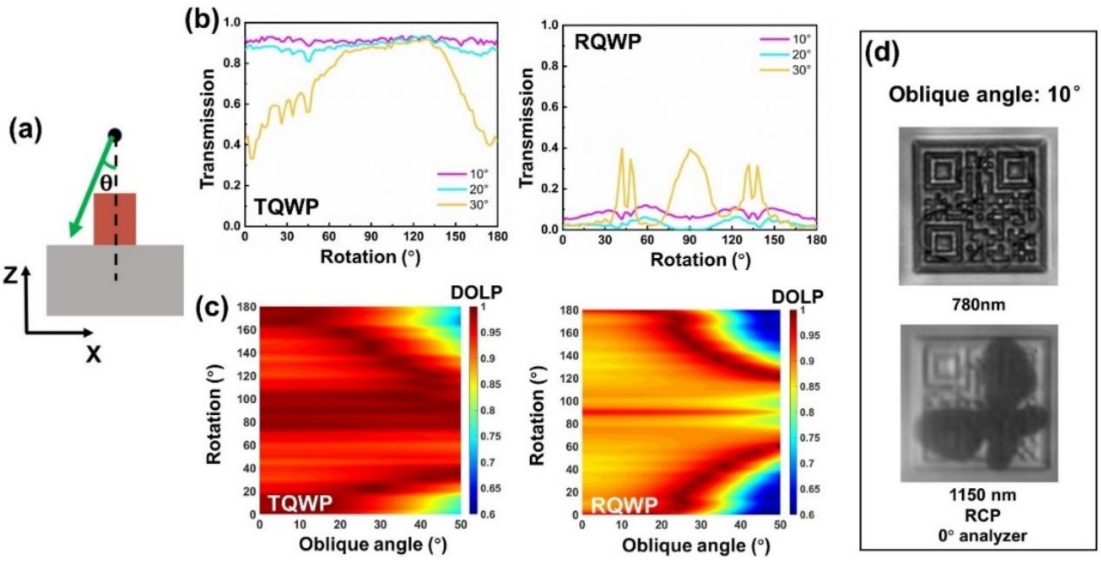

Figure S1. (a) Schematic illustrating oblique incidence. (b) The simulated transmission as a function of the TQWP and RQWP rotation angle under oblique incidence angles of 10°, 20°, and 30° for the wavelength of 800 nm. (c) The simulation results of DOLP as a function of the structure rotation angle and incidence angle at the wavelength of 1200 nm. (d) The experimentally obtained images for the wavelengths of 800 nm and 1200 nm, at an oblique angle of 10° .

Figure S1 provides the results of the meta-images under oblique incidence angles. Figure S1(a) first depicts the oblique incidence schematic. The transmissions as a function of the rotation angle of TQWP and RQWP are investigated for oblique incidence angles of 10°, 20°, and 30° for the wavelength of 800 nm (Figure S1(b)). Then, for the wavelength of 1200 nm, the corresponding DOLP are also examined as a function of the structure rotation angle and incidence angle (Figure S1(c)). The results demonstrate that the structures can only hold good performance when the incidence angle is less than 20°. We managed to acquire the images of the dual-binary metasurface sample measured at the incidence angle of 10°, where the QR code and butterfly patterns can be clearly seen as shown in Figure S1(d).

Note S2

Analysis on the effect of transmission difference on image quality

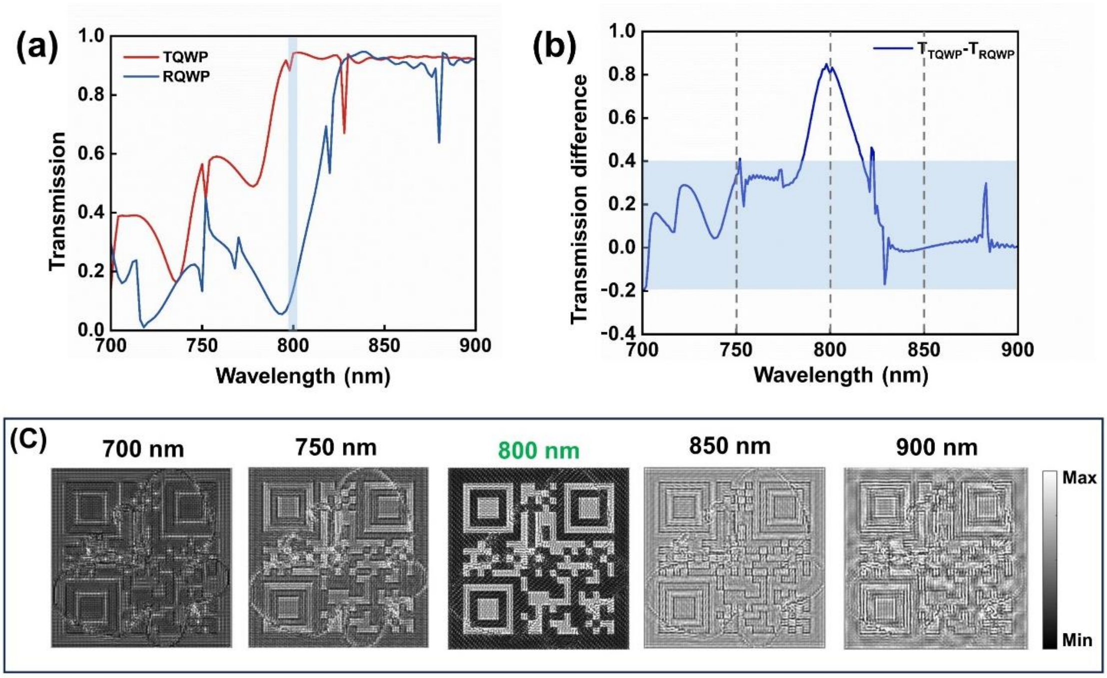

Figure S2. (a) The simulated transmission spectrum of TQWP and RQWP under RCP incidence from 700 nm to 900 nm. (b) The transmission difference between TQWP and RQWP under RCP incidence from 700 nm to 900 nm. (c) The simulated transmission light intensity profiles at five wavelengths including the designed one.

As shown in Figure S2, most values for the transmission difference of TQWP and RQWP drop below 40% in the wavelength range from 700 nm to 900 nm except that near the designed wavelength of 800 nm. Taking the meta-image at wavelength 750 nm as an example, the transmission difference of the two nanostructures is around 34%, and the image cannot be seen clearly. Instead of providing a specific intensity contrast value, we prefer to use the correlation coefficient (CC) to judge whether the meta-image is as desired or not. The CC is used to calculate the level of similarity between a target image and a simulation image [1]. The computational formula is in equation (1):

$$CC = \left(I_{\text{target}}, I_{\text{simulation}}\right) = \frac{Cov(I_{\text{target}}, I_{\text{simulation}})}{\sqrt{Cov(I_{\text{target}}, I_{\text{target}})}\sqrt{Cov(I_{\text{simulation}}, I_{\text{simulation}})}} \quad (1)$$

where I_{target} and $I_{\text{simulation}}$ are the intensity values of the target and simulation images, respectively, while Cov represents the covariance operation. In theory, if CC has a value higher than 0.6, it means the two images are strongly correlated, and we can define that the binary image is as-designed could be clearly seen. At the designed wavelength of 800 nm, the transmission difference is approximately 83%, and the corresponding CC value is 0.76. However, for other wavelengths like 750 nm, the CC value is only 0.34. The corresponding CC value at other wavelengths near the designed wavelength of 800 nm is also shown in Table 1.

Table 1. CC between the target image and simulation image at various wavelengths

Wavelength	700 nm	750 nm	800 nm	850 nm	900 nm
CC	-0.22	0.34	0.76	-0.19	-0.12

Note S3

Simulation analysis of the meta-image boundary where TQWP and RQWP are adjacently arranged

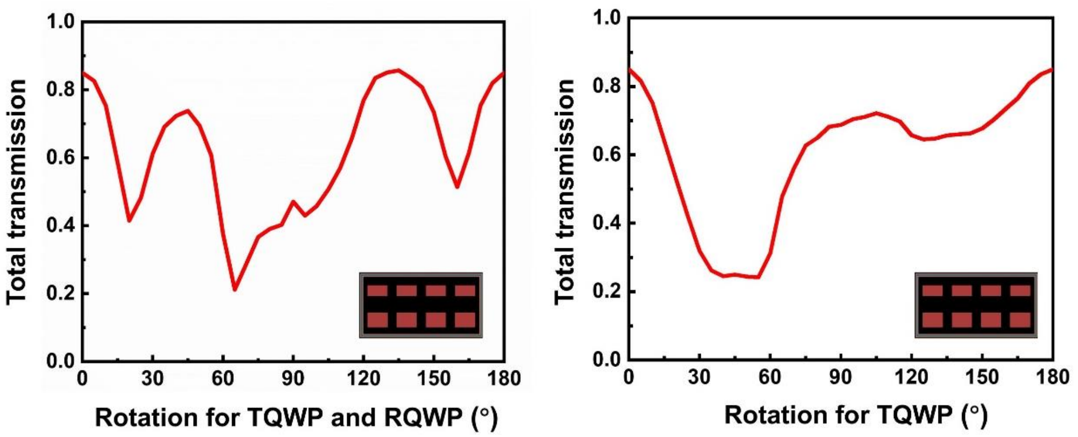

Figure S3. Simulated total transmission as a function of the nanostructure rotation angle when the TQWP and RQWP are adjacently arranged.

The image crosstalk mainly exists at the wavelength of 1200 nm. This implies that in addition to the image designed for the wavelength of 1200 nm, one will also see the image designed for the wavelength of 800 nm. As such, we investigate the boundary of the image designed for the wavelength of 800 nm where TQWP and RQWP are nearby, as indicated in Figure S3. On the other hand, the rotation angle determines the meta-image at wavelength of 1200 nm, therefore, in theory there should be no transmission changes as the rotation angle of TQWP and RQWP varies. However, it can be seen that when the two structures are adjacently placed, there are a lot of transmission fluctuations for the wavelength of 1200 nm. Meanwhile, the desired circular-to-linear polarization conversion functionality may also worsen in an unpredictable manner.

Note S4

Analysis of the metasurface under white light illumination

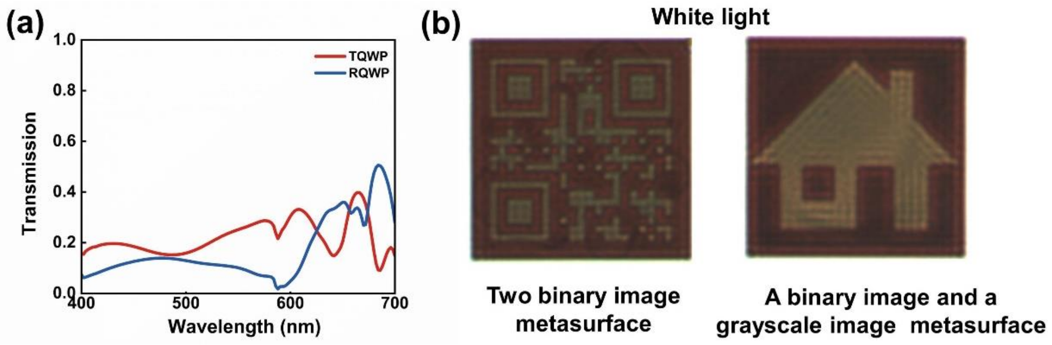

Figure S4. (a) Simulated transmission spectra of the two nanostructures (TQWP and RQWP) under natural white light illumination. (b) Optical images of the fabricated two metasurface samples.

Figure S4(a) depicts the visible transmission spectra of the two designed meta-atoms (TQWP and RQWP). The difference in transmission spectra implies that the two structures will display different colors. Figure S4(b) displays the corresponding experimental results. The appearance of the metasurface under white light illumination only resembles the image designed at 800 nm but the image designed at wavelength 1200 nm cannot be revealed.

Figure S5

The simulation results for the binary and grayscale meta-image metasurface under different incidence conditions.

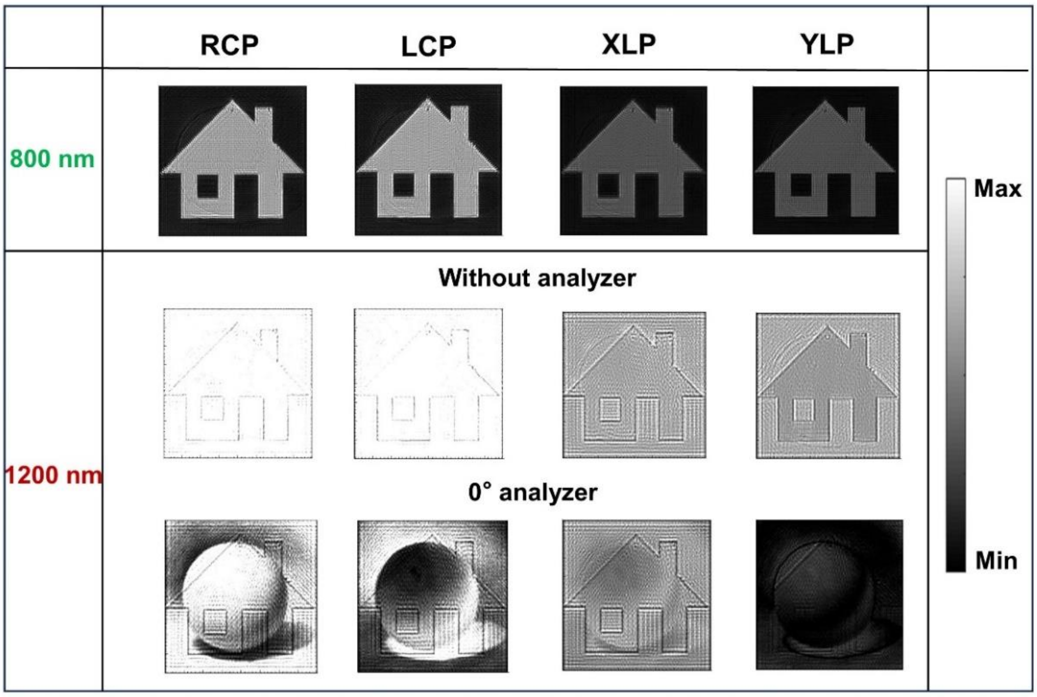

Figure S5. Simulated meta-images under different incidence conditions at wavelengths of 800 nm and 1200 nm.

Figure S6

The experimental setup for measuring the meta-image in dual-wavelength channel

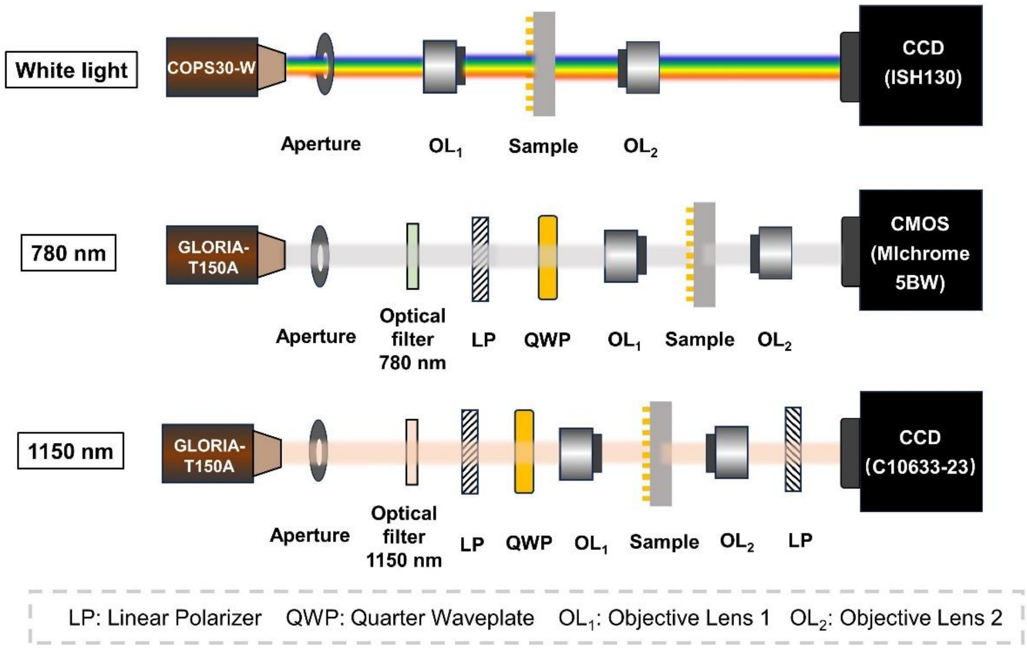

Figure S6. Measurement setup for acquiring the meta-images.

Reference

[1] J. Deng, F. Gao, P. Yuan, et al., "Bidirectional nanoprinting based on bilayer metasurfaces," *Opt. Express*, vol. 30, no. 1, pp. 377-388, 2022.
